# Antibody response and soluble mediator profile in the first six months following acute SARS-CoV-2 infection

**DOI:** 10.1038/s41598-023-43263-y

**Published:** 2023-10-30

**Authors:** Camila A Corsini, Priscilla S Filgueiras, Nathalie BF Almeida, Daniel AP de Miranda, Sarah VC Gomes, Adelina Junia Lourenço, Cecilia MF Bicalho, Jessica V de Assis, Raquel NH Amorim, Raphael A Silva, Raquel VR Vilela, Tulio M Lima, Daniel PB de Abreu, Renata GF Alvim, Leda R Castilho, Olindo A Martins-Filho, Dayane A Otta, Rafaella FQ Grenfell

**Affiliations:** 1 Diagnosis and Therapy of Infectious Diseases and Cancer, Oswaldo Cruz Foundation (FIOCRUZ), 1715 Augusto de Lima Avenue, Belo Horizonte, Minas Gerais 30190-002 Brazil; 2 Department of Pathology, College of Medicine, Federal University of Minas Gerais, 6627 Avenida Presidente Antônio Carlos, Belo Horizonte, Minas Gerais 31270-901 Brazil; 3 Hospital da Baleia, Benjamin Guimarães Foundation, 1464 Juramento Street, Belo Horizonte, Minas Gerais 30285-408 Brazil; 4 Cell Culture Engineering Laboratory, COPPE, Universidade Federal do Rio de Janeiro, 550 Pedro Calmon Avenue, Rio de Janeiro, Rio de Janeiro 21941-598 Brazil; 5 Grupo Integrado de Pesquisa em Biomarcadores, Oswaldo Cruz Foundation (FIOCRUZ), 1715 Augusto de Lima Avenue, Belo Horizonte, Minas Gerais 30190-002 Brazil; 6 Department of Infectious Diseases, College of Veterinary Medicine, University of Georgia, 501 DW Brooks Drive, Athens, GA 30602-7387 USA

**Keywords:** Immunology, Microbiology, Diseases, Medical research

## Abstract

The COVID-19 pandemic has caused a severe global health and economic crisis, with significant consequences for human mortality and morbidity. Therefore, there is an urgent need for more studies on the immune response to SARS-CoV-2 infection, both to enhance its effectiveness and prevent its deleterious effects. This study presents the chronology of antibodies during six months after infection in hospitalized patients and the kinetics of serum soluble mediators of the cellular response triggered by SARS-CoV-2. Samples and clinical data from 330 patients hospitalized at the Hospital da Baleia in Belo Horizonte, Brazil, who were suspected of having COVID-19, were collected at the time of hospitalization and during 6 months after infection. The immune response was analyzed by enzyme-linked immunosorbent assay (ELISA) and flow cytometry. There was a significant difference in IgM specific antibody titers from the 7th to 60th days after infection between COVID-19 negative and positive patients. Soon after 60 days after infection, antibody levels started to reduce, becoming similar to the antibody levels of the COVID-19 negative patients. IgG specific antibodies started to be detectable after 9 days of infection and antibody levels were comparatively higher in positive patients as soon as after 7 days. Furthermore, IgG levels remained higher in these patients during the complete period of 180 days after infection. The study observed similar antibody profiles between different patient groups. The soluble systemic biomarkers evaluated showed a decrease during the six months after hospitalization, except for CCL11, CXCL8, CCL3, CCL4, CCL5, IL-6, IFN-g, IL-17, IL-5, FGF-basic, PDGF, VEGF, G-CSF, and GM-CSF. The results indicate that IgM antibodies are more prominent in the early stages of infection, while IgG antibodies persist for a longer period. Additionally, the study identified that patients with COVID-19 have elevated levels of biomarkers after symptom onset, which decrease over time.

## Introduction

New scientific and clinical evidence is being evaluated regarding subacute and long-term effects after SARS-CoV-2 infection, which may affect multiple systems and lead to sequelae^1,2^. Reports from infected patients suggest residual effects of the infection, such as fatigue, dyspnea, chest pain, neurological disorders, arthralgia, and a decline in the quality of life^2–5^. Periods of intensive hospitalization, cell damage, intense innate immune response with exacerbated production of inflammatory cytokines, and a procoagulant state induced during infection by the virus may contribute to these observed sequelae^6–8^.

Studies show that COVID-19 is marked by dysregulation of myeloid cells^9^, and that critically ill patients have high serum concentrations of pro-inflammatory cytokines such as interleukins IL-6, IL-1β, IL-2, IL-8, IL-17, and tumor necrosis factor-α (TNF-α)^10^. However, further research is needed on the long-term behavior of these serum soluble mediators after infection with SARS-CoV-2, as data are scarce in the literature, especially in relation to unvaccinated COVID-19 survivors who presented moderate or severe illness requiring special care and hospitalization.

Our current understanding of SARS-CoV-2 immunity is based mainly on previous experiences with other corona viruses including SARS-CoV and MERS-CoV viral infections^11^ and needs to be supplemented with studies of patients who were infected and recovered from SARS-CoV-2 infection. To clarify the chronology of IgG and IgM antibodies and the kinetics of serum soluble mediators after acute infection by SARS-CoV-2, this work evaluated the immune response to the infection of unvaccinated and hospitalized patients, with suspected diagnosis of COVID-19. Molecular diagnoses were performed for COVID-19 confirmation. Blood samples were collected at the beginning of hospitalization and up to six months after the appearance of the first symptoms, determining the maintenance of the innate and humoral response.

## Methods

Three hundred and thirty unvaccinated patients who were suspected to have COVID-19 and were hospitalized at the Hospital da Baleia in Belo Horizonte, Brazil, between May 2020 and May 2021, were invited to participate in the study for a period of 6 months. Confirmation of COVID-19 was done by RT-qPCR after nasopharyngeal swab was collected between the third and seventh days after symptoms onset. Peripheral blood samples were collected from each patient on the first day of hospitalization until the fifteenth day after the onset of symptoms, every two days, followed by one collection per month for six months. Peripheral blood samples in the volume of 10 ml were collected by venipuncture, adhering to biosafety norms, and centrifuged at 3000 g/5 min for serum collection.

The clinical course of COVID-19 was classified based on the WHO classification^12^, and the severity was categorized as mild, moderate, or severe. Medical reports and records were used to monitor demographic information, primary symptoms, treatment protocols, molecular diagnosis results, comorbidities, and other individual information for each patient.

This study was analyzed and approved by the Human Research Ethics Committee of the Oswaldo Cruz Foundation and the National Research Ethics Committee (CONEP 30428720.3.0000.5091), and all the patients provided informed consent.

The serum samples from the whole study population were tested for specific IgM and IgG antibodies against the spike protein of SARS-CoV-2 (B.1). The antigen protein used was produced in stable recombinant HEK293 cells^13^, and antibody detection was measured by ELISA^14^. The antibodies used for the ELISA assays were both from the Sigma-Aldrich brand, Anti-Human IgM A0420 (Lot: 124M4811V) and Anti-Human IgG A0170 (Lot: 0181901), following the protocol by Grenfell et al., 2022^14^, considering a detection limit (cut off) of 0.35 and 0.1508 for IgM and IgG detection, respectively.

A high-throughput 27-plex Luminex assay (Bio-Rad) was used to quantify a range of immune soluble mediators in the sera of 61 participants confirmed for COVID-19 subgroup (Appendix 2), including chemokines (CXCL8, CCL11, CCL3, CCL4, CCL2, CCL5, CXCL10), pro-inflammatory cytokines (IL-1β, IL-6, TNF-α, IL-12, IFN-γ, IL-15, IL-17), regulatory cytokines (IL-1Ra, IL-4, IL-5, IL-9, IL-10, IL-13), and growth factors (FGF-basic, PDGF, VEGF, G-CSF, GM-CSF, IL-7, IL-2). Measurements were performed on a Bio-Plex 200 instrument (Bio-Rad). Samples were selected from these positive COVID-19 patients, collected between 30, 90, and 180 days after the onset of symptoms and hospital admission. As a control for this particular analysis, 37 serum samples from healthy volunteers (age from 22 to 54 years old, median 40 years) with negative RT-qPCR and no symptoms of COVID-19 were used. The selection of these samples was based on inclusion criteria for this group, with an effort to achieve age and gender matching with the patient group.

### Inclusion criteria for patients

Patients aged 18 or older, unvaccinated against COVID-19, with suspected or molecularly confirmed diagnosis of SARS-CoV-2, hospitalized and in isolation at Hospital da Baleia, Belo Horizonte, Brazil, from May 2020 to May 2021, who agreed to participate in the study and signed the informed consent form (ICF).

### Exclusion criteria for patients

Patients who did not agree to participate in the research and did not sign the ICF; Patients who received the primary COVID-19 vaccination protocol; Patients under 18 years of age.

### Inclusion criteria for healthy volunteers

Individuals aged 18 or older without known comorbidities and without a previous diagnosis of COVID-19 who agreed to participate in the study and signed the informed consent form (ICF).

### Exclusion criteria for healthy volunteers

Individuals under 18 years of age; presence of comorbidities and/or a previous diagnosis of COVID-19; Individuals who did not agree to participate in the study and did not sign the informed consent form (ICF).

Data analysis was performed using GraphPad Prism^®^ software. The results obtained with the quantification of antibodies titers were analyzed using Kruskal–Wallis test and Mann–Whitney normality tests with a significance level of p < 0.05. Signatures of soluble mediators were designed by converting the serum levels, originally expressed as continuous variables (pg/mL), into categorical data (percentage proportion) using the global median values as the cut off to identify the proportion of subjects with high levels of mediators. The proportion of subjects with increased levels (above the 50th percentile, gray zone) was underscored.

## Results

### Demographic data of the patients

A total of 330 unvaccinated patients with a suspected of COVID-19 who were hospitalized at the Hospital da Baleia in Belo Horizonte, Brazil, between May 2020 and May 2021, were separated into two groups based on RT-qPCR results: positive or negative for COVID-19 RT-qPCR. Among the 165 patients that were positive for COVID-19 (from 10 to 91 years, median age 59 years), 99 (60%) were male and 66 (40%) were female, as shown in Appendix 1. Hypertension (95, 57.6%), diabetes (53, 32.1%), chronic kidney disease (38, 23%) and cancer (24, 14.5%) were the most common comorbidities observed in this group. Sixteen (9.7%) presented no comorbidities. The main primary symptoms reported were dyspnea (89, 53.9%), fever (81, 49.1%), dry cough (41, 24.8%), myalgia (30, 18.2%), diarrhea (25, 15.2%), desaturation (23, 13.9%) and prostration (23, 13.9%). Regarding the medication protocol used, 107 patients (64.8%) made use of antibiotics, 70 (42.4%) nasal oxygen cannula, 37 (22.4%) corticosteroids, 23 (13.9%) antivirals and 14 (8.5%) anticoagulants. One hundred and thirty-three (80.6%) of the positive patients in the molecular diagnosis did not present clinical severity, however, 32 (19.4%) individuals required intensive treatment, and 17 (10.3%) of the patients evolved to death.

Among the 165 patients that were negative for COVID-19 (ranging from 1 to 88 years, median age 58 years), 88 (53.3%) were female and 77 (46.7%) were male. The most common comorbidities observed in this group were hypertension (84, 50.9%), chronic kidney disease (76, 46.1%), diabetes (46, 27.9%), and cancer (32, 19.4%). Seven (4.2%) of the hospitalized patients had no comorbidities. The primary symptoms reported were dyspnea (83, 50.3%), fever (66, 40%), dry cough (28, 17%), desaturation (27, 16.4%), myalgia (19, 11.5%), and odynophagia (17, 10.3%). Antibiotics (158, 95.8%), oxygen administration through nasal cannula (67, 40.6%), corticosteroids (33, 20%), anticoagulants (23, 13.9%), and antivirals (19, 11.5%) were the main resources used for medication during hospitalization. One hundred and forty-seven (89.1%) of the negative patients did not have clinical severity, while 18 (10.9%) required intensive treatment, and 10 (6.1%) of the patients died.

### Immunogenicity

**Figure 1:**
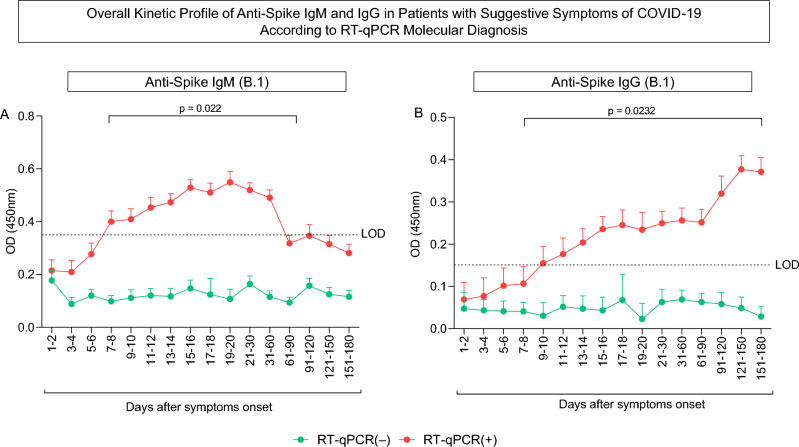
Overall kinetic profile of anti-Spike IgM and IgG in patients with suggestive symptoms of COVID-19 according to RT-qPCR molecular diagnosis. Overall kinetics are shown for (**A**) anti-Spike IgM antibodies and (**B**) anti-Spike IgG antibodies in negative and positive COVID-19 patients. The red and green lines represent the median and standard error of patient optical density throughout the six-month period. Limit of detection are demonstrated by the dotted limits respectively of 0.3500 and 0.1508 in the IgM and IgG ELISA. Statistical differences of Mann–Whitney and ANOVA with significance levels of p < 0.05 are presented during the analyzed period.

**Figure 2:**
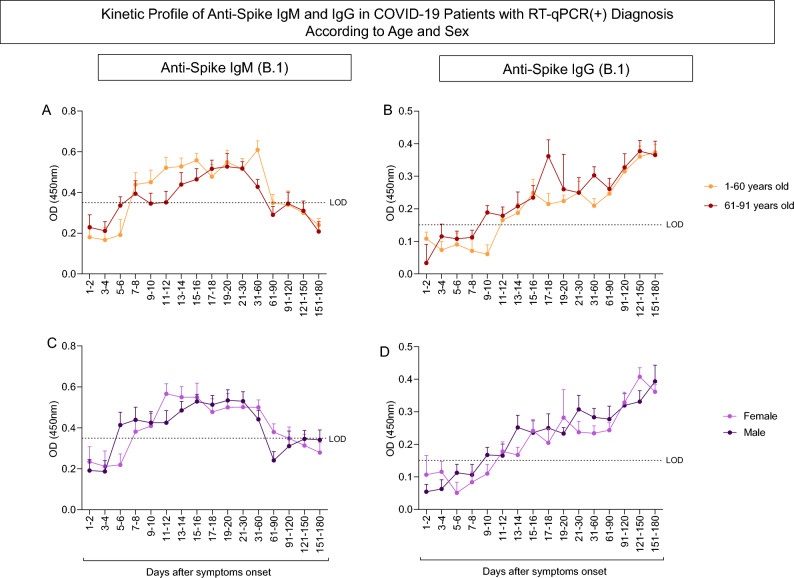
Kinetic profile of anti-Spike IgM and IgG in COVID-19 patients with RT-qPCR( +) diagnosis according to age and biological sex. Figure shows the kinetics of (**A**) IgM antibodies and (**B**) IgG antibodies for patients grouped by age, the kinetics of (**C**) IgM antibodies and (**D**) IgG antibodies of patients grouped by biological sex. The dotted lines represent the limit of detection (LOD) respectively of 0.3500 and 0.1508 in the IgM and IgG ELISA. Colored lines present the median and standard error of the patients’ optical densities during the evaluated period. Statistical difference by Mann–Whitney and ANOVA with significance levels of p < 0.05 are presented between the groups.

**Figure 3:**
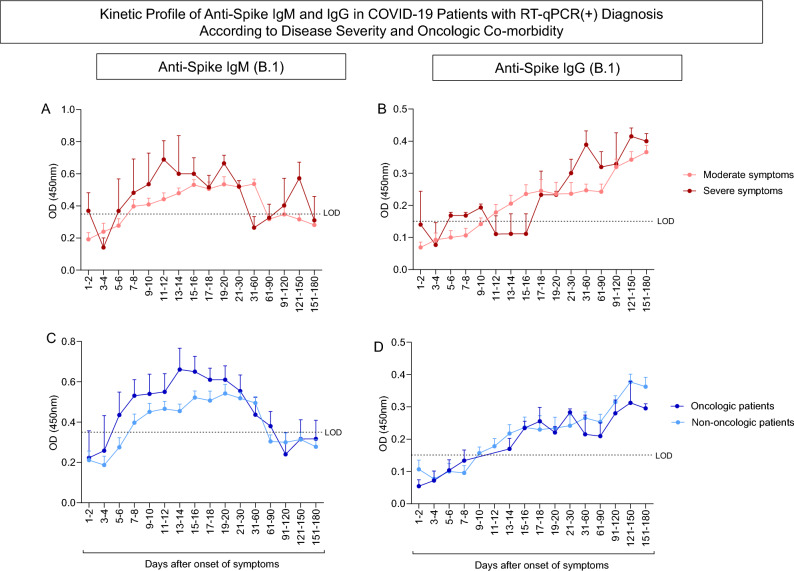
Kinetic profile of anti-Spike IgM and IgG in COVID-19 patients with RT-qPCR( +) diagnosis according to disease severity and oncologic co-morbidity. Figure shows the kinetics of (**A**) IgM antibodies and (**B**) IgG antibodies for patients grouped by COVID-19 severity, the kinetics of (**C**) IgM antibodies and (**D**) IgG antibodies of patients grouped by the presence or absence of oncologic co-morbidity. The dotted lines represent the limit of detection (LOD) respectively of 0.3500 and 0.1508 in the IgM and IgG ELISA. Colored lines present the median and standard error of the patients’ optical densities during the evaluated period. Statistical difference by Mann–Whitney and ANOVA with significance levels of p < 0.05 are presented between the groups.

In order to evaluate the longitudinal profile of IgM and IgG antibodies among negative and positive RT-qPCR patients, graphs using the median optical density (OD) from the first day to 6 months after the onset of symptoms were constructed. There was a significant difference (p = 0.022) in IgM specific antibody titers from the 7th to 60th days after infection between COVID-19 negative and positive patients (Fig. 1A). During this period, positive patients presented high levels of IgM. Soon after 60 days after infection, antibody levels started to reduce, becoming similar to the antibody levels of the COVID-19 negative patients. Differently, IgG specific antibodies started to be detectable after 9 days of infection and antibody levels were comparatively higher in positive patients as soon as after 7 days (p = 0.0232). IgG levels remained higher in these patients during the complete period of 180 days after infection (Fig. 1B). An evaluation of the IgM and IgG antibodies profile in the COVID-19 positive patients separately by age, biological sex, presence or absence of cancer, and by clinical severity of COVID-19 presented no differences between groups (Figs. 2 and 3).

**Figure 4:**
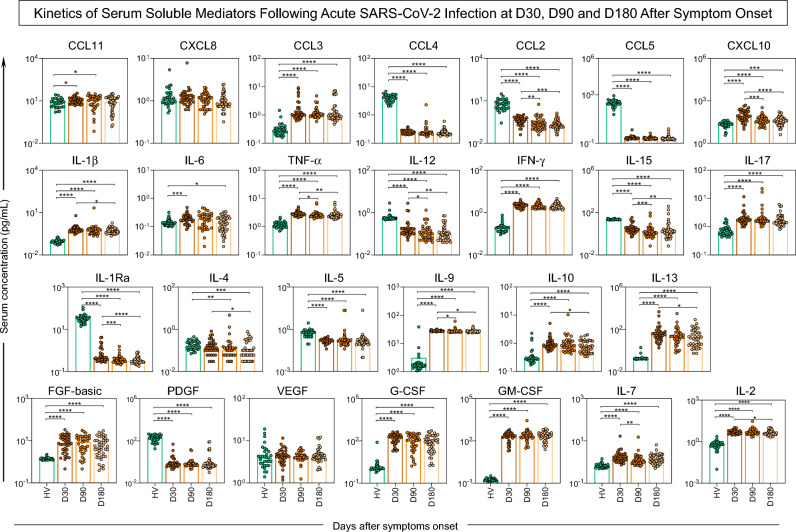
Kinetics of serum soluble mediators at 30 (D30), 90 (D90), and 180 days (D180) after symptom onset of acute SARS-CoV-2 infection as compared to the control group of healthy volunteers (HV). The biomarkers were individually presented in: chemokines (CXCL8, CCL11, CCL3, CCL4, CCL2, CCL5, CXCL10); pro-inflammatory cytokines (IL-1β, IL-6, TNF-α, IL-12, IFN-γ, IL-15, IL-17); regulatory cytokines (IL-1Ra, IL-4, IL-5, IL-9, IL-10, IL-13); and growth factors (basic FGF, PDGF, VEGF, G-CSF, GM-CSF, IL-7, IL-2). Statistical difference by Mann–Whitney and ANOVA with significance levels of p < 0.05 is denoted by (*) between groups.

When quantifying systemic soluble biomarkers using Luminex Bio-Plex Pro™ human cytokines in serum samples of COVID-19 positive patients at 30, 90, and 180 days after symptom onset, an important difference was observed between the healthy volunteers and positive patients. The mediators CCL3, CCL4, CCL2, CCL5, IL-12, IL-15, IL-1Ra, and PDGF were higher in healthy volunteers, while the mediators CCL11, CXCL10, IL-1b, IL-6, TNF-a, IFN-g, IL-17, IL-9, IL-10, IL-13, FGF-basic, G-CSF, GM-CSF, IL-7, and IL-2 were increased in COVID-19 positive patients (Fig. 4).

**Figure 5:**
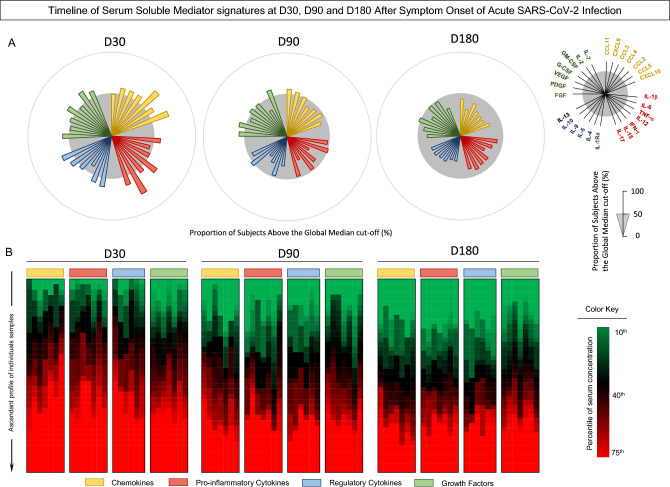
Timeline of serum soluble mediator signatures at 30 (D30), 90 (D90), and 180 days (D180) after symptom onset of acute SARS-CoV-2 infection. The biomarkers were individually presented in subpanels and displayed in classes: chemokines (CXCL8, CCL11, CCL3, CCL4, CCL2, CCL5, CXCL10); pro-inflammatory cytokines (IL-1β, IL-6, TNF-α, IL-12, IFN-γ, IL-15, IL-17); regulatory cytokines (IL-1Ra, IL-4, IL-5, IL-9, IL-10, IL-13); and growth factors (basic FGF, PDGF, VEGF, G-CSF, GM-CSF, IL-7, IL-2). In (**A**) overall signature of mediators; (**B**) heatmap constructs.

The kinetics of the evaluated biomarkers among positive COVID-19 patients showed a significant reduction in most soluble mediators from the first evaluation after 30-days of SARS-CoV-2 infection to 180 days after symptom onset, except for CCL11, CXCL8, CCL3, CCL4, CCL5, IL-6, IFN-g, IL-17, IL-5, FGF-basic, PDGF, VEGF, G-CSF, and GM-CSF (Figs. 4 and 5).

Considering the establishment of the general signature of serum soluble mediators after acute SARS-CoV-2 infection, it was identified that most patients had biomarkers levels above the global median 30 days after the onset of symptoms. However, these levels were reduced in relation to the global median after six months of infection (Fig. 5A).

## Discussion

The clinical manifestations of individuals diagnosed with COVID-19 can be predominantly characterized by a set of flu-like symptoms, such as shortness of breath, fever, cough, and myalgia, which were reported by most patients in this study as well as in other previously published research^15–17^. The development of more severe symptoms of COVID-19 and the need for hospitalization are mainly associated with elderly patients (> 60 years old) with one or more pre-existing comorbidities^18–21^. The presence of underlying chronic diseases in patients infected with SARS-CoV-2 such as hypertension, diabetes, and chronic kidney disease increases the risk of developing moderate to severe COVID-19 with the need for medical intervention up to 10 times compared to individuals without any comorbidity, which corroborates the results of this research, where the vast majority of hospitalized patients who required intensive treatment were elderly with at least one pre-existing comorbidity^17,22,23^.

Regarding the adaptive immune response, previous studies have shown that circulating antibodies against SARS-CoV reach their peak 4 months after the onset of the disease and persist for at least 2 years^24^. In addition, neutralizing antibodies against Middle East respiratory syndrome coronavirus remain detectable for at least 34 months after the outbreak^25^.

In a study conducted by Hsueh et al. (2004)^26^ to evaluate the chronological evolution of IgM, IgA, and IgG antibodies after SARS-CoV infection using immunofluorescent antibody assays, it was found that seroconversion for IgG occurred on average at 10 days, simultaneously or one day before IgM and IgA, which had an average seroconversion of 11 days. It was also described that patients whose lung condition was not severe had IgG profiles like those patients who later developed severe respiratory failure requiring ventilatory support^26^.

Regarding COVID-19, most individuals infected with SARS-CoV-2 seroconvert within 5 to 15 days after the onset of symptoms, with approximately 90% seroconversion by the tenth day^27–31^. In addition, estimates of seroconversion in the production of anti-SARS-CoV-2 Spike protein antibodies can vary from 91 to 99% in large studies^32,33^, where IgA, IgM, and IgG antibodies develop within a few days in infected individuals^29,31,34^, as observed in this study, where COVID-19 positive patients had a seroconversion of IgM and IgG antibodies, respectively, on the seventh and ninth day after symptom onset. Data from two large studies with more than a thousand participants reported that IgG titers against SARS-CoV-2 are well maintained up to 4 months after symptom onset^32,33^. In this study, patients had high levels of IgG throughout the 6-month period analyzed. Furthermore, neutralizing antibodies can be detected in most infected patients from 4 days after symptom onset, except in severe patients who may present these antibodies up to 3 weeks later^35^.

Long et al. (2020)^27^ validated a magnetic chemiluminescence enzyme immunoassay for the detection of specific anti-SARS-CoV-2 antibodies in 285 infected patients. They found that IgM-type antibody levels varied widely among patients, as did IgG. Furthermore, they observed that antibody titers, in general, in the group of patients with severe COVID-19 were higher than those in the group that did not show clinical severity of the disease^27^. KOWITDAMRONG and collaborators (2020)^36^ analyzed the correlation between the level of antibodies and age in the severe group and no significant correlation was found. Patients who had the severe form of the disease in the present study did not show such a difference during the antibody chronology performed, nor did other evaluated groups, such as the difference between age group, biological sex, and presence or absence of neoplasia.

The host immune response to COVID-19 determines susceptibility to the progression of infection as well as being a major determinant of recovery orchestrated by coordinated cell an antibody responses^11^. Furthermore, data concerning the durability of immunity to SARS-CoV-2 infection can assist in the continued development of successful vaccines and therapeutics for control of the infection. When quantifying systemic soluble biomarkers of surviving COVID-19 positive patients, it was observed that chemokines, pro-inflammatory cytokines, regulatory cytokines, and growth factors showed a decrease during the six months of follow-up, except for some particular biomarkers: CCL11, CXCL8, CCL3, CCL4, CCL5, IL-6, IFN-g, IL-17, IL-5, FGF-basic, PDGF, VEGF, G-CSF, and GM-CSF.

The cytokines produced during an infection by SARS-CoV-2 exert antiviral and inflammatory activities and are also directly associated with the pathological processes of the disease, such as cell death and immunothrombosis^37^. The multisystemic immune dysregulation observed in patients with moderate or severe COVID-19 includes an increase in various pro-inflammatory cytokines, such as interleukin-6 (IL-6), tumor necrosis factor-alpha (TNFα), interferon-gamma (IFNγ), and IL-17, compared to healthy volunteers^10,37–40^, as also observed in this study.

While an effective immune response against SARS-CoV-2 requires the involvement of both innate and adaptive immune cells, patients with COVID-19 present some variations in their immune response, such as a reduction in lymphocyte count (lymphopenia) and an increase in the neutrophil/lymphocyte ratio, which are considered disease markers^41–43^.

To better clarify the relationship between changes in the peripheral immune system and the severity of COVID-19, Walter et al. (2022)^44^ quantified the levels of inflammatory mediators during acute COVID-19. They observed that infected patients presented an increase in myeloperoxidase, interleukins IL-12, IL-6, IL-10, and IL-8, accompanied by a reduction in IL-17A and nitric oxide levels. IL-10 levels ≥ 14 pg/ml were strongly related to worse disease outcomes, with a sensitivity of 78.3% and specificity of 79.1%. Among the cytokines evaluated, IL-6 was the most related to a worse prognosis in patients with COVID-19, as it was increased in most patients, especially in those with more severe disease^44^. Exacerbated IL-6 responses can lead to a systemic pro-inflammatory response, as this interleukin is involved in several mechanisms such as the acute phase response, inflammation, proliferation of B and T cells, hematopoiesis, and neutrophil chemotaxis^45^.

The irregular innate immune response triggered by SARS-CoV-2 is associated with the development of post-infection sequelae. Published studies have shown that some inflammatory cytokines, including IL-6, TNF-α, and IL-1β, are elevated in patients with Long Covid^46^. Additionally, cytokines such as IFN-β and IFN-λ1 may remain elevated for 8 months after acute infection in these patients compared to recovered individuals. Furthermore, cytokines IL-6, IFN-γ, IFN-β, PTX3, IFN-γ, and IFN-λ2/3 was associated with Long Covid with 78.5 to 81.6% accuracy^47^.

In view of the results of this work and other previously discussed studies, the innate and adaptive immune response during COVID-19 is still controversial, which may be closely related to several other specific clinical characteristics of each patient. Therefore, understanding the heterogeneous manifestations of this disease and exploring the relationships between these phenomena and immunity is a priority. Additionally, it is crucial to understand the duration of memory and protective immunity to SARS-CoV-2 after infection or vaccination.

## Conclusion

In conclusion, this study showed that COVID-19 positive patients presented high levels of anti-SARS-CoV-2 spike IgG antibodies throughout the 6-month period analyzed, with no difference between age, biological sex, and presence or absence of neoplasia groups over time. Soluble biomarkers were increasingly released at the beginning of the infection followed by a decrease during the six months of follow-up on some specific chemokines, pro-inflammatory and regulatory cytokines, and growth factors as: CCL11, CXCL8, CCL3, CCL4, CCL5, IL-6, IFN-g, IL-17, IL-5, FGF-basic, PDGF, VEGF, G-CSF, and GM-CSF. These findings contribute to understanding of the immune response to COVID-19 and may have implications for the development of effective treatments and vaccines. However, further research is needed to fully understand the long-term immune response and potential implications for reinfection and vaccination strategies.

### Supplementary Information

Supplementary Information 1.

Supplementary Information 2.

## Data Availability

The datasets used and/or analysed during the current study available from the corresponding author on reasonable request.
