# Supplementary material for: Antibody response and soluble mediator profile in the first six months following acute SARS-CoV-2 infection
Source: Sci Rep. 2023 Oct 30;13:18606. doi: 10.1038/s41598-023-43263-y (PMC10616118; doi:10.1038/s41598-023-43263-y)
Supplement: Supplementary file 1 — Supplementary Information 1. [file 41598_2023_43263_MOESM1_ESM.pdf]

Appendix 1 - Demographic data and characteristics of 330 patients admitted to the Hospital da Baleia, Belo Horizonte, Brazil, from May 2020 to May 2021 with suspected COVID-19: detectable and non-detectable RT-qPCR.

Epidemiological data	Detectable RT-qPCR (N=165)		Not detectable RT-qPCR (N=165)		TOTAL (N=330)	
	N	%	N	%	N	%
Age, years						
≤20	2	1.2	6	3.6	8	2.4
21-30	0	0.0	5	3.0	5	1.5
31-40	16	9.7	19	11.5	35	10.6
41-50	27	16.4	30	18.2	57	17.3
51-60	42	25.5	33	20.0	75	22.7
61-70	43	26.1	44	26.7	87	26.4
71-80	21	12.7	17	10.3	38	11.5
≥81	14	8.5	11	6.7	25	7.6
Biological sex						
Male	99	60.0	77	46.7	176	58.7
Female	66	40.0	88	53.3	154	46.7
Comorbidities						
Hypertension	95	57.6	84	50.9	179	54.2
Diabetes	53	32.1	46	27.9	99	30.0
Chronic kidney disease	38	23.0	76	46.1	114	34.5
Cancer	24	14.5	32	19.4	56	17.0
Smoking	23	13.9	24	14.5	47	14.2
Obesity	22	13.3	17	10.3	39	11.8
Alcoholism	15	9.1	10	6.1	25	7.6
Heart disease	14	8.5	18	10.9	32	9.7
Pulmonary fibrosis	13	7.9	15	9.1	28	8.5
Asthma	10	6.1	12	7.3	22	6.7
Dyslipidemia	9	5.5	5	3.0	14	4.2
Hypothyroidism	8	4.8	6	3.6	14	4.2
Stroke	3	1.8	5	3.0	8	2.4
Rheumatoid arthritis	2	1.2	1	0.6	3	0.9
Alzheimer	2	1.2	1	0.6	3	0.9
Crack user	1	0.6	4	2.4	5	1.5
HIV	1	0.6	5	3.0	6	1.8
Hepatitis C	1	0.6	1	0.6	2	0.6
No comorbidities	16	9.7	7	4.2	23	7.0
Primary symptoms						
Dyspnea	89	53.9	83	50.3	172	52.1
Fever	81	49.1	66	40.0	147	44.5
Dry cough	41	24.8	28	17.0	69	20.9
Myalgia	30	18.2	19	11.5	49	14.8

Desaturation	23	13.9	27	16.4	50	15.2
Prostration	23	13.9	13	7.9	36	10.9
Diarrhea	25	15.2	10	6.1	35	10.6
Anosmia	17	10.3	7	4.2	24	7.3
Odynophagia	17	10.3	17	10.3	34	10.3
Headache	16	9.7	7	4.2	23	7.0
Asthenia	14	8.5	13	7.9	27	8.2
Coryza	12	7.3	14	8.5	26	7.9
Oligoproductive cough	7	4.2	10	6.1	17	5.2
Productive cough	7	4.2	11	6.7	18	5.5
Chest pain	5	3.0	7	4.2	12	3.6
Tachydyspnea	3	1.8	0	0.0	3	0.9
Nausea	3	1.8	3	1.8	6	1.8
Mental confusion	2	1.2	2	1.2	4	1.2
Edema	1	0.6	4	2.4	5	1.5
Clinical severity						
Non-severe	0	0	0	0	0	0
Severe	133	80.6	147	89.1	280	84.8
Critical	32	19.4	18	10.9	50	15.2
Medication protocol						
Antibiotic	107	64.8	158	95.8	265	80.3
Use of nasal oxygen cannula	70	42.4	67	40.6	137	41.5
Corticosteroid	37	22.4	33	20.0	70	21.2
Antiviral	23	13.9	19	11.5	42	12.7
Anticoagulant	14	8.5	23	13.9	37	11.2
Mechanical ventilation	12	7.3	4	2.4	16	4.8
Antacids	9	5.5	2	1.2	11	3.3
Bronchodilator	5	3.0	7	4.2	12	3.6
Analgesic and antipyretic	4	2.4	12	7.3	16	4.8
Diuretic	4	2.4	4	2.4	8	2.4
Vermifuge	4	2.4	1	0.6	5	1.5
Neuroleptic	1	0.6	0	0.0	1	0.3
Antimalarial	0	0.0	1	0.6	1	0.3
Clinical outcome						
Discharged from hospital	148	89.7	155	93.9	303	91.8
Death	17	10.3	10	6.1	27	8.2
