# Supplementary material for: Antibody response and soluble mediator profile in the first six months following acute SARS-CoV-2 infection
Source: Sci Rep. 2023 Oct 30;13:18606. doi: 10.1038/s41598-023-43263-y (PMC10616118; doi:10.1038/s41598-023-43263-y)
Supplement: Supplementary file 2 — Supplementary Information 2. [file 41598_2023_43263_MOESM2_ESM.pdf]

Appendix 2 - Demographic data and characteristics of 61 patients admitted to the Hospital da Baleia, Belo Horizonte, Brazil, from May 2020 to May 2021: Participants confirmed for COVID-19 subgroup (High-throughput 27-plex Luminex assay).

Participants confirmed for COVID-19 subgroup (N=61)

High-throughput 27-plex Luminex assay

Epidemiological data	N	%
Age. years		
≤20	1	1.64
21-30	0	0.00
31-40	7	11.48
41-50	8	13.11
51-60	14	22.95
61-70	34	55.74
71-80	7	11.48
≥81	4	6.56
Biological sex		
Male	31	50.82
Female	30	49.18
Comorbidities		
Hypertension	42	68.85
Diabetes	24	39.34
Chronic kidney disease	17	27.87
Cancer	8	13.11
Smoking	7	11.48
Obesity	7	11.48
Alcoholism	6	9.84
Heart disease	6	9.84
Hypothyroidism	4	6.56
Asthma	3	4.92
No comorbidities	3	4.92
Dyslipidemia	2	3.28

Pulmonary fibrosis	1	1.64
Stroke	1	1.64
Alzheimer	1	1.64
HIV	1	1.64
Hepatitis C	1	1.64
Primary symptoms		
Dyspnea	38	62.30
Fever	30	49.18
Dry cough	18	29.51
Diarrhea	12	19.67
Myalgia	10	16.39
Anosmia	10	16.39
Desaturation	9	14.75
Odynophagia	8	13.11
Prostration	7	11.48
Headache	7	11.48
Coryza	5	8.20
Tachydyspnea	3	4.92
Nausea	3	4.92
Asthenia	2	3.28
Oligoproductive cough	1	1.64
Productive cough	1	1.64
Clinical severity		
Non-severe	0	0.00
Severe	61	100.00
Critical	0	0.00
Clinical outcome		
Discharged from hospital	61	100.00
Death	0	0.00
